# A High-Resolution Crystal Structure of a Psychrohalophilic α–Carbonic Anhydrase from *Photobacterium profundum* Reveals a Unique Dimer Interface

**DOI:** 10.1371/journal.pone.0168022

**Published:** 2016-12-09

**Authors:** Vijayakumar Somalinga, Greg Buhrman, Ashikha Arun, Robert B. Rose, Amy M. Grunden

**Affiliations:** 1 Department of Plant and Microbial Biology, North Carolina State University, Raleigh, NC, United States of America; 2 Department of Molecular and Structural Biochemistry, North Carolina State University, Raleigh, NC, United States of America; Griffith University, AUSTRALIA

## Abstract

Bacterial α–carbonic anhydrases (α-CA) are zinc containing metalloenzymes that catalyze the rapid interconversion of CO_2_ to bicarbonate and a proton. We report the first crystal structure of a pyschrohalophilic α–CA from a deep-sea bacterium, *Photobacterium profundum*. Size exclusion chromatography of the purified *P*. *profundum* α–CA (PprCA) reveals that the protein is a heterogeneous mix of monomers and dimers. Furthermore, an “in-gel” carbonic anhydrase activity assay, also known as protonography, revealed two distinct bands corresponding to monomeric and dimeric forms of PprCA that are catalytically active. The crystal structure of PprCA was determined in its native form and reveals a highly conserved “knot-topology” that is characteristic of α–CA’s. Similar to other bacterial α–CA’s, PprCA also crystallized as a dimer. Furthermore, dimer interface analysis revealed the presence of a chloride ion (Cl^-^) in the interface which is unique to PprCA and has not been observed in any other α–CA’s characterized so far. Molecular dynamics simulation and chloride ion occupancy analysis shows 100% occupancy for the Cl^-^ ion in the dimer interface. Zinc coordinating triple histidine residues, substrate binding hydrophobic patch residues, and the hydrophilic proton wire residues are highly conserved in PprCA and are identical to other well-studied α–CA’s.

## Introduction

Carbonic anhydrases (CA; carbonate hydro-lyase EC 4.2.1.1) are metallo-enzymes that catalyze the reversible hydration of carbon dioxide (CO_2_) to bicarbonate [1]. CA’s are ubiquitous in nature and are classified into 6 different groups (α, β, γ, δ, ζ and η) [2]. The α-CA’s are primarily found in vertebrates and are the only type of carbonic anhydrase expressed in mammals [1]. The human α–CA is one of the most extensively studied carbonic anhydrase because of its pharmacological relevance [1]. Recently, several α–CA’s have been identified and characterized in prokaryotes [3]. The beta carbonic anhydrase (β–CA) was first identified in plants [4], and ever since β–CA’s have been identified in other organisms including photosynthetic bacteria where they play an important role in the inter-conversion of CO_2_ to bicarbonate essential for photosynthesis [5]. The gamma carbonic anhydrase (γ-CA) was initially thought to be restricted to archaea, although recently several eubacterial γ-CA’s have been identified and characterized [1]. The delta and zeta carbonic anhydrases (δ-CA and ζ-CA) are found only in algae and diatoms while the recently identified sixth class of carbonic anhydrase (η-CA) is found in the pathogenic protozoan, *Plasmodium falciparum* [2]. All carbonic anhydrases are metalloenzymes with either zinc, iron, or cadmium coordinated via three histidine residues in the active site [1]. The reaction mechanism of carbonic anhydrases has been extensively studied in human α-carbonic anhydrase II (hCAII); and with a turnover rate of 10^6^ s^-1^, it is one of the most catalytically efficient isoforms of human carbonic anhydrase [6]. The hydration of CO_2_ to bicarbonate occurs via a two-step ping-pong mechanism starting with the nucleophilic attack of the carbon of the CO_2_ group by the zinc-bound hydroxide [7]. The bicarbonate thus formed is displaced by a water molecule that directly coordinates with zinc. The regeneration of the hydroxide occurs via the intra and intermolecular proton transport achieved by a series of hydrophilic amino acids in the active site cavity. The final step in this process is the transfer of the proton to the bulk solvent via a histidine residue that is highly conserved in many carbonic anhydrases [6, 7]. This two-step reaction is essential for the efficiency of carbonic anhydrases and made possible by a series of hydrophilic residues in the active site. The CO_2_ is held in place by hydrophobic residues proximal to the catalytic metal ion. This arrangement of active site residues is highly conserved among all classes of carbonic anhydrases [8].

Bacterial CA’s play varied physiological roles ranging from pathogenesis to carbon fixation. Recently interest has grown in the prokaryotic CA’s due to their potential applications in industrial carbon capture and in biofuel production. Carbonic anhydrases from extremophiles are ideal for these applications due to their propensity to withstand extremes of conditions that are common in industrial settings. To date, only a few carbonic anhydrases from extremophiles have been characterized, and as such, most of these carbonic anhydrases are from either thermophiles or halophiles. In this report, we describe the first crystal structure of a psychrophilic α–carbonic anhydrase from *Photobacterium profundum* (*P*. *profundum*), a psychrohalophile isolated from deep-sea sediment. We also show that PprCA is functional and retains the structural core that is conserved in all carbonic anhydrases. Furthermore, our work reveals a chloride ion in the dimer interface which is unique to PprCA and has not been observed in any other α–CA’s.

## Materials and Methods

### Cloning, over-expression and purification of *P*. *profundum* α-carbonic anhydrase

*Photobacterium profundum* SS9 chromosomal DNA was purchased from the American Type Culture Collection (ATCC) (USA). Gene specific primers were designed to amplify the α–carbonic anhydrase gene (KEGG gene entry: PBPRA3376) excluding the region coding for the signal peptide. PCR amplification using primers Ppr_alpha P1 (GGA ATT C CAT ATG GCT GAA TGG AGT TAT ACT GGC G) and Ppr_alpha P2 (CCG CTC GAG TTA TTC TAA GAT CAG GCG CGC) (restriction enzyme sites are underlined) resulted in a 657 bp product that was purified using a Qiagen gel extraction kit (Qiagen, MD). The purified PCR product was digested with Nde I and Xho I restriction enzymes and was ligated to a pET28a vector digested with the same enzymes. Ligations were transformed into *E*. *coli* XL_Blue (Stratagene, CA) competent cells for propagation. Ligated plasmids were purified from the transformants and were sequenced to confirm the presence of the carbonic anhydrase gene.

The plasmid was transformed into *E*. *coli* BL21(DE3) for overexpression. Cells were grown in LB broth containing 35 μg/ml Kanamycin at 37°C, OD_600_ = 0.6., Subsequently, the temperature was reduced to 20°C, and CA gene expression was induced with 0.5 mM Isopropyl-β-thiogalactoside (IPTG) and grown overnight. Cells were harvested by centrifugation (4000 rpm, 20 min at 4°C) and stored at -20°C.

Cell pellets were re-suspended in Buffer A (20 mM Tris pH 7.5, 500 mM NaCl, 30 mM imidazole, 1 mM benzamidine and 1 mM phenyl-methylsulfonly fluoride [PMSF]) and lysed with a French press pressure cell (Aminco international, USA). The cell lysate was centrifuged for 30 min at 12,000 rpm at 4°C and the supernatant was passed through a 0.45-μM syringe filter before being loaded on to a 5 ml Ni-Sepharose HisTrap HP column (GE healthcare, Pittsburgh, PA pre-equilibrated with Buffer A. The protein was eluted with Buffer A with a linear gradient of imidazole and the eluted protein was buffer-exchanged in 20 mM Tris pH 7.5, 150 mM NaCl buffer over-night at 4°C. As a final step, the protein was purified with a Hiprep 16/60 Sephacryl S-200 (GE healthcare, Pittsburgh, PA) gel-filtration column. For crystallization, the purified protein was concentrated to 12.5 mg/ml by ultrafiltration using an Amicon Centrifugal Filter, MWCO 10 kDa (Millipore, MA).

### Protonography

The in-gel carbonic anhydrase assay was performed as described in De Luca et al, 2015 [9]. Briefly, the *P*. *profundum* α–carbonic anhydrase (PprCA) was mixed with Laemmli loading buffer without 2-mercaptoethanol. The sample separation was carried out on a 12% SDS gel. Subsequently, the gel was soaked in 2.5% Triton X-100 for 1 hour on a shaker and washed twice with 100 mM Tris, pH 8.2 containing 10% isopropanol for 10 min. After washing, the gel was incubated in 0.1% bromothymol blue in 100 mM Tris, pH 8.2 for 30 min. To visualize carbonic anhydrase activity, the gel was immersed in CO_2_-saturated ddH_2_O which was prepared by bubbling CO_2_ for 3 hours.

### Crystallization of *P*. *profundum* α-carbonic anhydrase

Vapor diffusion sitting-drop crystallization screens for *P*. *profundum* α–carbonic anhydrase (PprCA) were set-up in 96-well 3-drop crystallization plates (Hampton Research). One microliter of protein solution (7.5 mg/ml, 10 mg/ml and 12.5 mg/ml) was mixed with 1 μl of crystallization screen conditions using a crystallization robot (Phoenix, Art Robbins Instruments). A single large crystal formed after two weeks in NeXtal Protein Complex Suite containing 0.1M HEPES pH 7 and 15% (w/v) PEG 4000. The crystal was harvested, soaked in mother liquor containing 15% PEG 400 as cryoprotectant. The soaked crystals were flash frozen and stored in liquid nitrogen until data collection.

### Data collection and refinement

Data were collected at the Southeast Regional Collaborative Access Team (SER-CAT) 22-BM) beamline at the Advanced Photon Source, Argonne National Laboratory. A single 1.0 Angstrom wavelength data set was collected at 100 K, with 1.0-degree oscillation per frame and a crystal to detector distance of 150mm (Table 1). The crystal diffracted to a resolution of 1.5 Å. The raw data was integrated and scaled using the HKL2000 software package [10]. The data was scaled in space group P3(2)21, with unit cell parameters of a = b = 60.461 Å, c = 95.464 Å and α, β, γ = 120°.

**Table 1:** Data collection and refinement statistics.

Crystal	PprCA
Wavelength (Å)	1
Resolution range (Å)	50–1.5 (1.53–1.5)
Space group	P3_2_21
Unit cell	60.5Å 60.5Å 95.5Å 90° 90° 120°
Total reflections	260731
Unique reflections	32971
Multiplicity	7.6 (6.4)
Completeness (%)	99.99 (99.94)
Mean I/sigma (I)	17.26 (2.94)
Wilson B-factor	11.82
R-merge	0.256 (0.408)
R-work	0.1389 (0.1910)
R-free	0.1678 (0.2559)
Number of atoms	3732
macromolecules	1722
Zn, Cl ions	1,1
Water	342
Protein residues	217
RMS (bonds)	0.009
RMS (angles)	1.36
Ramachandran favored	98
Ramachandran outliers	0
Clash score	3.24
Average B factor	16.10
macromolecules	14.30
ligands	10.40
solvent	25

The structure of PprCA was solved by molecular replacement using the automated molecular replacement module of Phaser. The search model was a monomer of CA from *Thermovibrio ammonificans* (PDB code: 4C3T), which has 46.67% sequence identity with PprCA. For the initial model, all alternate conformations and hydrogen atoms were removed and all B-factors were set to 30.00. Phaser found a single solution for a monomer in the asymmetric unit with a Matthews coefficient of 1.88 and a solvent content of 34.60%. The single solution had a LLG score of 316 and a R_work_ of 54%.

Residues were mutated to the PprCA sequence during autobuilding. The Phenix autobuild routine successfully placed and built 214 residues with a final R_work_ of 23% and an R_free_ of 25%. Further refinement included manual rebuilding in Coot and refinement in Phenix [11]. Water molecules were added manually into Fo-Fc difference density contoured at 3.0σ. Water molecules were discarded if no significant 2Fo-Fc electron density contoured at 1.0σ was present in subsequent rounds of refinement. For the final rounds of refinement, alternate conformations were added, hydrogens were added and the final model was refined using a TLS model which consisted of four groups: residues 14–35, residues 36–145, residues 146–163 and residue 164–230.

An anomalous difference map was calculated from the reflection data that were rescaled for anomalous scaling using the HKL2000 software package [10]. Based on the rescaled data, the anomalous difference map was generated in Phenix [11] using the solved model for phase information. The overall anomalous signal was 0.148 calculated from intensity differences of 41,735 Bijvoet pairs. The resulting anomalous difference map was visualized in Coot and images were created in PyMol using contours of 8.0σ for zinc site and 3.5 for chloride and sulfur positions.

### Molecular dynamic (MD) simulations

The stability of the chloride ion in the dimer interface was investigated by MD using Gromacs 4.6.5 and the Gromos53a6 force field. The starting model was constructed from the protein X-ray structure with the following modifications: removal of alternate conformations, inclusion of the symmetry-related molecule to generate the dimer, inclusion of one zinc ion in the active site of each monomer and with or without the chloride ion buried in the dimer interface. The model was placed in the center of a 12nm^3^ cubic box of 55307 SPC model water molecules and 0.1mM NaCl with 72 sodium and chloride ions plus 17 extra sodium ions to neutralize the charge of the starting model. Steepest descent energy minimization converged to F_max_ < 1000 in 638 steps with a Potential Energy of -2.8 X 10^6^ kJ/mole. The system was equilibrated with 100ps of NVT dynamics at 288 K with 2 femto seconds (fs) time steps using the v-rescale thermostat, followed by 100ps of NPT dynamics at 1.0 bar using the Parrinello-Rahman barostat with positional restraints. The potential energy stabilized at -2.5 X 10^6^ kJ/mole after NVT simulation and NPT simulation. The initial production run consisted of 1 nano second (ns) simulation time with a 2 fs time step at 288 K and 1.0 bar with H-bonds constrained and no positional restraints. The production run was then extended to 5ns.

## Results and Discussion

### Overall structure of PprCA

**Fig 1:**
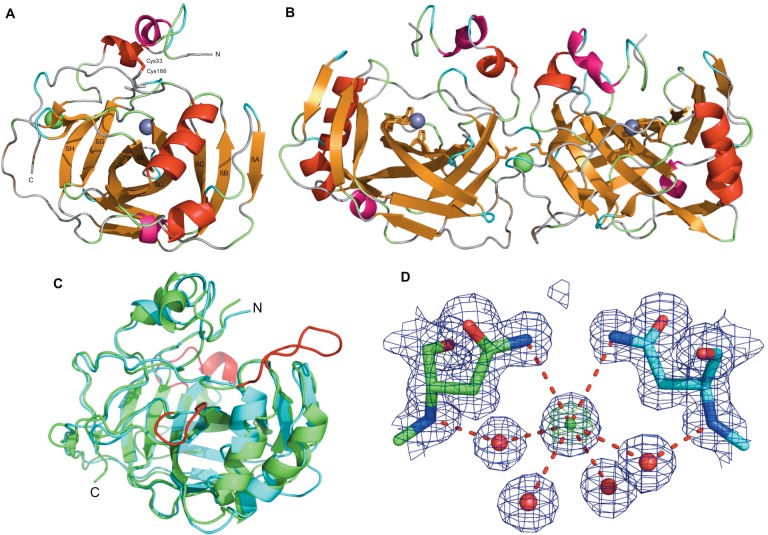
Overall structure of PprCA and structural comparison with prokaryotic and human α–CA. **(A)** Cartoon representation of the PprCA monomer with central ten-stranded β-sheets colored gold. An intra-molecular disulfide bond that stabilizes the monomer is shown as stick model. Catalytic zinc ions are shown as gray spheres while the chloride ion is shown in green **(B)** Cartoon representation of the PprCA dimer with α-helices and β-sheets colored according to structural conservation. The dimer interface chloride ion is shown in green. The active site zinc ions are shown as grey spheres. **(C)** Comparison on PprCA monomer with hCAII. hCAII is shown in green while PprCA is shown in blue. Surface loops regions of hCAII are colored red. **(D)** Chloride ion coordination by four water molecules and Asn 178/ND2 from Chain A (green) and Chain B (blue). 2Fo-Fc electron density contoured at 8.0σ (green) and 1.0σ (blue).

The final model was refined at 1.5 Angstrom resolution to R_work_ of 13.9% and R_free_ of 16.8%. The final structure is completely built from residues 25–241 excluding the 23 amino acid N-terminal signal peptide sequence. The electron density for the N-terminal hexa-histidine tag and thrombin cleavage site was not visible presumably due to the highly disordered nature of this region. This high-resolution structure has excellent geometry, with 98.17% of residues in the favored portion of the Ramachandran plot and the rest in the allowed portion of the Ramachandran plot. Only 0.53% of the side chains are not in energetically unfavorable rotamers, with no Cβ outliers. Most of the residues are well ordered, with an average B-factor of 14.3 Å^2^. In addition to protein atoms, the final model includes 1 zinc ion in the active site, 1 chloride ion and 342 waters (Table 1). PprCA has the characteristic carbonic anhydrase fold consisting of a ten-stranded, twisted β-sheet that is antiparallel except for 2 pairs of parallel β-strands and a few short α-helices (Fig 1A). The core β-sheet contains the important catalytic zinc bound to the histidine triad residues (Fig 1A). Structural comparison between PprCA and the human hCAII, a well-studied, representative member of the α–carbonic anhydrase family shows several large deletions corresponding to surface loops in hCAII (Fig 1C). The largest of these deletion is 11 residues long, while two more deletions, 10 and 6 residues long were also identified in PprCA (Fig 1C). Similar deletions were also reported for NgCA [3] and SspCA [12]. The large loop deletion located on the upper edge of the active site contains a 5-residue helical region that has been shown to be critical for ligand recognition in human α–carbonic anhydrase [13] while the shorter loop deletions are on the surface of the protein and have been reported to have no apparent effect on the catalytic activity of α–carbonic anhydrase [12]. In SspCA, the result of the larger loop deletion is the enlargement of the active site cavity [12]. The absence of the large loop in PprCA may result in similar increase in active site cavity and is likely in all the other α–carbonic anhydrases that lack the large loop region.

### PprCA crystallizes as a dimer with a unique chloride ion in the dimer interface

The biological unit of most bacterial CA's are dimers or tetramers though some seem to function as monomers [12]. The PprCA crystal contains a monomer in the asymmetric unit. The dimer formed by the crystallographic 2-fold axis buries 751 Å^2^ of total surface area and superimposes well with other known bacterial CA dimers. The PprCA dimer was superimposed with 11 currently available structures of bacterial CA dimers from mesophiles, thermotolerant bacteria, thermophiles and hyperthermophiles. The overall rmsd of the monomers was 0.752, and of the dimers was 1.041, suggesting the PprCA dimer in the crystal structure may be biologically relevant.

The dimer interface of PprCA consists mainly of symmetrical polar contacts between hydrophilic side chains, water molecules and the interfacial chloride ion. Residues involved in interfacial side chain hydrogen bonds include Arg176, Arg191, Glu188, Asn178 and Asn38. The sidechain of Asn178 also interacts directly with the interface chloride ion. The only interfacial main chain hydrogen bonds occur across the symmetrical beta bridge formed between residues 49–51. A chloride ion was built into 8σ 2Fo-Fc electron density at the center of the PprCA dimer interface (Fig 1D). At this Sigma contour, the only other visible electron density is for the active site zinc ion and sulfur atoms from cysteine and methionine side chains. A Cl- ion was chosen because it was the only anion present in the protein buffer and crystallization condition. The ion is completely buried in the dimer interface between electropositive surfaces created by Arg176 and Arg226 from each chain. The Cl- ion is hexa-coordinated to the amide side chains of Asn178, symAsn178 (3.43 Å) and 4 water molecules (3.1–3.26 Å) (Fig 1D), close to the ideal Cl–O distance of 3.2 Å.

Although the CA dimers superimpose with an overall rmsd of 0.752, the presence of a chloride ion is unique in prokaryotic CA structures. In other dimer structures this volume is occupied by ordered water molecules. The residues in the dimer interface are highly conserved from psychrophiles to thermophiles, except for Asn178, the residue interacting with the Cl ion, which is a serine in thermophiles TaCA (4C3T), SspCA (4G7A), an alanine in mesophiles NgCA (1KOP) and an asparagine in HpαCA (4XFW). HpαCA has Asn187 at the equivalent location as Asn178 in PprCA; however, there is no chloride ion in the dimer interface of HpαCA. Instead, there are two water molecules that form an H-bond network connecting the backbone amide of Asn187 from chain B to the side chain carbonyl oxygen of Asn187 from chain A. Whereas the interactions in PprCA are symmetrical across the dimer interface, this is not the case in HpαCA.

To further investigate the nature of the ions in the dimer interface and the active site, the raw reflection data was re-scaled in scalepack (HKL-2000 software package) to treat Friedel pairs separately and used to calculate an anomalous difference map. As expected, this map revealed a significant peak centered for the zinc ion (S1 Fig). The anomalous difference map also showed significant peaks near the putative dimer interface chloride ion (S1 Fig). These peaks appear to be anisotropic, but are similar in sigma level, size and shape to peaks found near known sulfur atoms (S1 Fig). In our experience, these results are consistent with the relative anomalous signal expected from zinc, chloride and sulfur atoms from data collected at a wavelength of 1.0 Å.

### The chloride ion is stable in MD simulations

To explore the potential structural significance of the chloride ion buried in the dimer interface, identical MD simulations were run with (Cl+) and without (Cl-) the chloride ion present. Overall, simulation parameter averages for potential energy, kinetic energy, temperature, volume, density and enthalpy are the same for both Cl+ and Cl- simulations. The Cα- r.m.s.d. increases from 1.0–2.5 Å over the first 500 picoseconds (ps) and then stabilizes at 2.5 Å over the final 500 ps in both simulations. The dimer interface chloride ion is coordinated to the side chain nitrogen of Asn178 from both chains throughout the entire simulation, similar to the protein x-ray structure with a ND-Cl distance of 3.0–4.0 Å for both chains. The MD simulation contained 72 chloride ions. Although several low occupancy chloride ion binding sites were detected in electropositive pockets on the protein surface, the only ion present at 100% occupancy was the dimer interface chloride ion (S2 Fig). An identical analysis of chloride ion binding sites in the Cl- trajectory showed that instead of forming a stable coordination with the dimer interface chloride, N178 is more flexible and forms multiple, short lived inter-subunit H-bonds with either N178 or N38 from the opposite chain (S2 Fig).

One of the distinct features of PprCA is the presence of a chloride ion in the dimer interface. To our knowledge, none of the other bacterial α–CA’s characterized contain an ion in the dimer interface. MD simulations show that the chloride ion is stably coordinated with two Asn178 side chains from both monomers of PprCA in the dimer interface (Fig 1D). The chloride ion in the dimer interface of PprCA may represent halophilic adaptation by PprCA to a high salt environment. Similar chloride ions have been reported in the dimer interface of tetrameric malate dehydrogenase (Hm MalDH) from a halophilic archaeon, *Haloarcula marismortui* [14]. The chloride ions of Hm MalDH form charge interactions with Lysine, Arginine and Aspartic acid residues from both the monomers [14]. Numerous studies have shown that Lys and Arg residues are common in complex salt bridges since these residues serve as critical connectors due to their interaction geometry in case of Arg or due to the charged nature of the interacting residue (Lys) [14]. Coordination of ions at protein interfaces may represent a common strategy by halophilic enzymes to stabilize oligomeric complexes.

### The active site is highly conserved in PprCA

**Fig 2:**
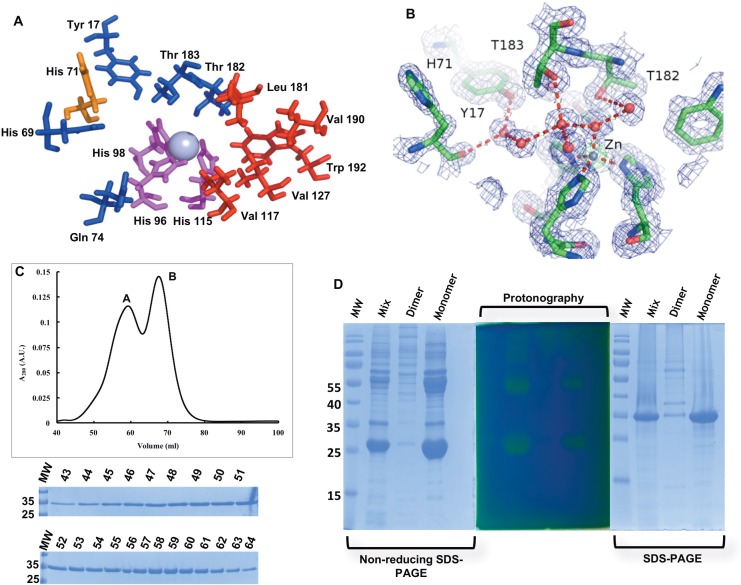
Cartoon representation of the PprCA active site and oligomerization status of PprCA. (**A**) The highly conserved active site residues of PprCA showing Zinc ion in the center of the active site (gray sphere). The hydrophobic (Val117, Val127, Leu181, Val190 and Trp192), the hydrophilic (Tyr17, Asn69, Gln74, Thr182 and Thr183), and zinc coordinating residues (His96, His98 and His115) are similar to other well characterized α–CA’s. (**B**) Active site water network involved in proton transfer is shown as red spheres. 2Fo-Fc electron density contoured at 8.0σ (green) and 1.0σ in blue. (**C**) Purified PprCA was analyzed on a Hiprep 16/60 Sephacryl S-200 size-exclusion column that was equilibrated with 20 mM Tris pH 7.5 and 150 mM NaCl. Peaks A and B correspond to dimer and monomer, respectively. The elution fractions were analyzed by SDS-PAGE and stained with Coomassie Blue. A single ~35 kDa band was observed for both dimeric and monomeric PprCA. MW–Molecular-weight marker, lanes 43 to 51 (elution fractions representing PprCA dimer) and lanes 52 to 64 (elution fractions representing PprCA monomer). (**D**) Purified PprCA was separated under both reducing and non-reducing conditions on a SDS-PAGE. Lanes were loaded with PprCA from monomer fraction, dimer fraction and from a sample containing a mixture of monomers and dimers. The gels were stained either by Coomassie blue or subjected to protonography. Protonography was performed according to De Luca et al, 2015 [9]. The gel was incubated in CO_2_ enriched water for 5 to 15 seconds at room temperature. Appearance of distinctive yellow bands in gels subjected to protonography indicates both monomeric (~27 kDa) and dimeric (~58 kDa) PprCA is catalytically active.

The PprCA active site is very similar to other α–carbonic anhydrases with all three catalytically essential regions highly conserved (S3 Fig). The zinc ion in active site of PprCA is coordinated by three histidine residues (His 96, His 98 and His 115) and a water molecule, denoted as Zinc-bound water (Fig 2A). This water molecule, along with the active site hydrophilic residues (Tyr17, Asn69, Gln74, Thr182 and Thr183), play an important role in this catalytically essential proton transfer mechanism) [15]. The only difference in these 5 residues long hydrophilic patch in α-carbonic anhydrases is the central Gln74 which is replaced by other hydrophilic residues: Asparagine in hCAII [16], or Lysine in SSpCA [12], and TaCA [17]. Substrate binding in α–carbonic anhydrases is facilitated by the hydrophobic patch (Val117, Val127, Leu181, Val190 and Trp192) that is highly conserved in all the known α–CA’s [16]. The hydrophobic patch in PprCA is identical to its closest homolog, NgCA [3] and the well-studied prototypical α–CA, hCAII [16]. Several studies have shown that the residues of the hydrophobic patch are critical for substrate binding and any perturbations in these residues results in the loss of catalytic activity [8]. The penultimate step in the reversible hydration of carbon dioxide to bicarbonate is the release of a proton to the bulk solvent [15]. This step is facilitated by a conserved histidine residue (His71) (Fig 2A and 2B) located on the edge of the active site cavity [15]. The position of this histidine residue in PprCA is identical to that of the other characterized α–CA‘s indicating that the reaction mechanism of CO_2_ hydration is essentially similar in PprCA.

### Size exclusion chromatography and protonography show PprCA is active as a monomer and dimer

Analysis of the oligomeric status of PprCA by SEC shows two distinct peaks (Fig 2C) indicating that PprCA in solution is found in both monomeric and dimeric forms. To date, all bacterial α–CA’s characterized are either dimers [3,12] or in some cases tetramers [17]. The dimeric arrangement of α–CA’s seems to be characteristic of bacterial α–CA’s since the well-studied human α–CA is a monomer. Analysis of oligomeric status of PprCA by SEC shows two distinct peaks (Fig 2C) indicating that PprCA is stable in solution in both monomeric and dimeric forms. The exact reason why PprCA occurs as a mix of monomers and dimers in solution remains unclear and needs further investigation.

Protonography is a reliable and quick method for analyzing CO_2_ hydratase activity of carbonic anhydrases in SDS gels [9]. Protonography has been successfully used as a rapid method for detecting CO_2_ hydratase activity of carbonic anhydrases from purified protein or and even can detect the activity of carbonic anhydrase from whole cell extracts by detecting the pH change associated with formation of the bicarbonate product [9]. Protonography is a relatively new, reliable and quick method for analyzing CO_2_ hydratase activity of carbonic anhydrases in SDS gels [9]. The carbonic anhydrases separated by SDS-gels are stained by a pH indicator, bromothymol blue, which changes color from blue to yellow when the pH drops from 8.2 to 6.8. This color change is due to the release of protons during the catalytic conversion of CO_2_ to bicarbonate and occurs in the gel where carbonic anhydrase is localized [9]. Analysis of PprCA by protonography revealed that these proteins exhibit characteristic CO_2_ hydratase activity as evidenced by the formation of yellow bands (Fig 2D) and furthermore, the use of non-reducing conditions for the separation of PprCA for protonography reveals two distinct bands with molecular weights consistent with the monomeric and dimeric forms of PprCA (Fig 2D). The presence of two distinct peaks in SEC along with functional bands corresponding to monomeric and dimeric PprCA revealed by protonography shows that these proteins are found as mixed oligomers in solution.

## Conclusion

Carbonic anhydrases are ubiquitous in nature, and due to their importance in physiology, they have been extensively characterized. Identification of carbonic anhydrases in bacteria, especially in extremophiles has renewed interest in these enzymes due to their potential for industrial application. In this paper, we report the first crystal structure of a unique α–CA purified from a psychrohalophile, *P*. *profundum*. The purified PprCA crystallized as a dimer and retained the highly conserved structural core characteristic of α–CA’s. Dimer analysis revealed a chloride ion in the interface that is unique to PprCA. Interestingly, PprCA in solution was shown to exist as a mix of monomers and dimers by SEC and non-reducing SDS-PAGE. Furthermore, protonography revealed that both monomers and dimers of PprCA retained catalytic activity. The residues and the water network of the catalytic core of PprCA is essentially similar to other characterized α–CA’s indicating that PprCA retains the CO_2_ hydration mechanism that is identical in all the other carbonic anhydrases. More work is underway to completely understand the oligomerization status and the role of chloride ion in the dimer interface of psychrohalophilic α–CA’s.

## Accession Number

Coordinates and structure factors for PprCA have been deposited in the Protein Data Bank with the accession number 5HPJ.

## Supporting Information

S1 Fig Anomalous Fourier difference map at sulfur, chloride and zinc positions in PprCA. A. An anomalous difference map at sulfur positions of the intra-molecular disulfide formed by Cys33 and Cys186. 2Fo-Fc electron density contoured at 1.0σ is shown in blue mesh. Anomalous difference density contoured at 3.5σ is shown in green mesh. B. Active site of PprCA with the zinc ion shown as a grey sphere and water molecules shown as red spheres. Anomalous difference density is contoured at 8.0σ and shown as a green mesh surrounding the zinc ion. C. Dimer interface chloride ion with interacting Asn residues. The chloride ion is shown as a green sphere. Anomalous difference density is contoured at 3.5σ and is shown as a green mesh.
(DOCX)

S2 Fig Molecular dynamics simulation for chloride occupancy in Ppr_alpha. A-C: Average distances for potential H-bond partners between Asn178 and Asn38 in CL- 5ns MD simulation. A: The H-bond between Chain A Asn178ND2 and Chain B Asn38OD1 is lost at 2.5ns and reforms at 4.5ns. B: The H-bond between Chain A Asn38OD1 and Chain B Asn178ND2 is lost at 0.5ns and 4.2ns. C: The H-bond between Chain A Asn178OD1 and Chain B Asn178ND2 is not present except for a brief moment at 0.5ns and 4.4ns and appears to correspond with the loss of the H-bond shown in B. D: Average coordination distance for Asn178 to the dimer interface chloride ion in Cl+ 5ns MD simulations. D: The coordination distance between Asn178ND2 and the dimer interface chloride ion fluctuates stably from 3.2 Angstroms to 3.8 Angstroms for most of the simulation.
(DOCX)

S3 Fig Multiple sequence alignment of Ppr_alpha with α–carbonic anhydrases. 4C3T (*Thermovibrio ammonicians*), 1KOP (*Neisseria gonorrhoeae*), 4G7A (*Sulfurihydrogenibium yellowstonense*), 4XFW (*Helicobacter pylori*) and 1CA2 (*Homo sapiens*). Cysteine residues involved in intramolecular disulfide bond formation are shown in green, the penultimate histidine residue required for proton transfer is shown in yellow. Three histidine residues required for zinc co-ordination are shown in red.
(DOCX)
