# Supplementary material for: A High-Resolution Crystal Structure of a Psychrohalophilic α–Carbonic Anhydrase from Photobacterium profundum Reveals a Unique Dimer Interface
Source: PLoS One. 2016 Dec 9;11(12):e0168022. doi: 10.1371/journal.pone.0168022 (PMC5148590; doi:10.1371/journal.pone.0168022)
Supplement: S1 Fig — A. An anomalous difference map at sulfur positions of the intra-molecular disulfide formed by Cys33 and Cys186. 2Fo-Fc electron density contoured at 1.0σ is shown in blue mesh. Anomalous difference density contoured at 3.5σ is shown in green mesh. B. Active site of PprCA with the zinc ion shown as a grey sphere and water molecules shown as red spheres. Anomalous difference density is contoured at 8.0σ and shown as a green mesh surrounding the zinc ion. C. Dimer interface chloride ion with interacting Asn residues. The chloride ion is shown as a green sphere. Anomalous difference density is contoured at 3.5σ and is shown as a green mesh. (DOCX) [file pone.0168022.s001.docx]

**Supporting Information**

**Title: A high-resolution crystal structure of a psychrohalophilic α–carbonic anhydrase from *Photobacterium profundum* reveals a unique dimer interface**

**Author affiliation:** Vijayakumar Somalinga^1^, Greg Buhrman^2^, Ashikha Arun^1^, Robert B. Rose^2^ and Amy M. Grunden^1,3^

^1^Department of Plant and Microbial Biology, North Carolina State University, Raleigh, NC, U.S.A.

^2^Department of Molecular and Structural Biochemistry, North Carolina State University, Raleigh, NC, USA

**S1 Fig**: **Anomalous Fourier difference map at the sulfur, chloride and zinc positions in PprCA.** A. Anomalous difference map at sulfur positions of the intra-molecular disulfide formed by Cys33 and Cys186. 2Fo-Fc electron density contoured at 1.0σ is shown in blue mesh. Anomalous difference density contoured at 3.5σ is shown in green mesh. B. Active site of PprCA with the zinc ion shown as a grey sphere and water molecules shown as red spheres. Anomalous difference density is contoured at 8.0σ and shown as a green mesh surrounding the zinc ion. C. Dimer interface chloride ion with interacting Asn residues. The chloride ion is shown as a green sphere. Anomalous difference density is contoured at 3.5σ and is shown as a green mesh.

**S1 Fig: Anomalous Fourier difference map for cysteine, zinc and chloride ions in PprCA.**
