# Supplementary material for: A High-Resolution Crystal Structure of a Psychrohalophilic α–Carbonic Anhydrase from Photobacterium profundum Reveals a Unique Dimer Interface
Source: PLoS One. 2016 Dec 9;11(12):e0168022. doi: 10.1371/journal.pone.0168022 (PMC5148590; doi:10.1371/journal.pone.0168022)
Supplement: S2 Fig — A-C: Average distances for potential H-bond partners between Asn178 and Asn38 in CL- 5ns MD simulation. A: The H-bond between Chain A Asn178ND2 and Chain B Asn38OD1 is lost at 2.5ns and reforms at 4.5ns. B: The H-bond between Chain A Asn38OD1 and Chain B Asn178ND2 is lost at 0.5ns and 4.2ns. C: The H-bond between Chain A Asn178OD1 and Chain B Asn178ND2 is not present except for a brief moment at 0.5ns and 4.4ns and appears to correspond with the loss of the H-bond shown in B. D: Average coordination distance for Asn178 to the dimer interface chloride ion in Cl+ 5ns MD simulations. D: The coordination distance between Asn178ND2 and the dimer interface chloride ion fluctuates stably from 3.2 Angstroms to 3.8 Angstroms for most of the simulation. (DOCX) [file pone.0168022.s002.docx]

**Supporting Information**

**Title: A high-resolution crystal structure of a psychrohalophilic α–carbonic anhydrase from *Photobacterium profundum* reveals a unique dimer interface**

**Author affiliation:** Vijayakumar Somalinga^1^, Greg Buhrman^2^, Ashikha Arun^1^, Robert B. Rose^2^ and Amy M. Grunden^1,3^

^1^Department of Plant and Microbial Biology, North Carolina State University, Raleigh, NC, U.S.A.

^2^Department of Molecular and Structural Biochemistry, North Carolina State University, Raleigh, NC, USA

**S2 Fig: Molecular dynamics simulation for chloride occupancy in PprCA.** A-C: Average distances for potential H-bond partners between Asn178 and Asn38 in CL- 5ns MD simulation. A: The H-bond between Chain A Asn178ND2 and Chain B Asn38OD1 is lost at 2.5ns and reforms at 4.5ns. B: The H-bond between Chain A Asn38OD1 and Chain B Asn178ND2 is lost at 0.5ns and 4.2ns. C: The H-bond between Chain A Asn178OD1 and Chain B Asn178ND2 is not present except for a brief moment at 0.5ns and 4.4ns and appears to correspond with the loss of the H-bond shown in B. D: Average coordination distance for Asn178 to the dimer interface chloride ion in Cl+ 5ns MD simulations. D: The coordination distance between Asn178ND2 and the dimer interface chloride ion fluctuates stably from 3.2 Angstroms to 3.8 Angstroms for most of the simulation.

**Figure S2: Molecular dynamics simulation for chloride occupancy in PprCA.**
