# Supplementary material for: A High-Resolution Crystal Structure of a Psychrohalophilic α–Carbonic Anhydrase from Photobacterium profundum Reveals a Unique Dimer Interface
Source: PLoS One. 2016 Dec 9;11(12):e0168022. doi: 10.1371/journal.pone.0168022 (PMC5148590; doi:10.1371/journal.pone.0168022)
Supplement: S3 Fig — 4C3T (Thermovibrio ammonicians), 1KOP (Neisseria gonorrhoeae), 4G7A (Sulfurihydrogenibium yellowstonense), 4XFW (Helicobacter pylori) and 1CA2 (Homo sapiens). Cysteine residues involved in intramolecular disulfide bond formation are shown in green, the penultimate histidine residue required for proton transfer is shown in yellow. Three histidine residues required for zinc co-ordination are shown in red. (DOCX) [file pone.0168022.s003.docx]

**Supporting Information**

**Title: A high-resolution crystal structure of a psychrohalophilic α–carbonic anhydrase from *Photobacterium profundum* reveals a unique dimer interface**

**Author affiliation:** Vijayakumar Somalinga^1^, Greg Buhrman^2^, Ashikha Arun^1^, Robert B. Rose^2^ and Amy M. Grunden^1,3^

^1^Department of Plant and Microbial Biology, North Carolina State University, Raleigh, NC, U.S.A.

^2^Department of Molecular and Structural Biochemistry, North Carolina State University, Raleigh, NC, USA

**S3 Fig: Multiple sequence alignment of PprCA with α–carbonic anhydrases.** 4C3T (*Thermovibrio ammonicians*), 1KOP (*Neisseria gonorrhoeae*), 4G7A (*Sulfurihydrogenibium yellowstonense*), 4XFW (*Helicobacter pylori*) and 1CA2 (*Homo sapiens*). Cysteine residues involved in intramolecular disulfide bond formation are shown in green, the penultimate histidine residue required for proton transfer is shown in yellow. Three histidine residues required for zinc co-ordination are shown in red.

**Figure S3: Multiple sequence alignment of α–carbonic anhydrases**

Ppr_alpha --EWSYTG-EHGTEHWGD---SFATCAEGVNQTPIDINQT---TQAELAPLHLDYEGQ-V 50

4C3T --HWGYSG-SIGPEHWGDLSPEYLMCKIGKNQSPIDINSA-DAVKACLAPVSVYYVSD-A 55

1KOP HTHWGYTG-HDSPESWGNLSEEFRLCSTGKNQSPVNITET---VSGKLPAIKVNYKPS-M 55

4G7A --EWSYEG-EKGPEHWAQLKPEFFWCKL-KNQSPINIDKK-YKVKANLPKLNLYYKTAKE 55

4XFW --KWDYKNKENGPHRWDKLHKDFEVCKSGKSQSPINIEHY-YHTQDK-ADLQFKYAASKP 56

1CA2 SHHWGYGK-HNGPEHWHK---DFPI-AKGERQSPVDIDTHTAKYDPSLKPLSVSYDQATS 55

.* * . . * . .: *:*::* . : . *

Ppr_alpha TELVNNGHTIQANLTG-KNTLTVDG----KTFELKQFHFHT------PSENYLKGKQYPL 99

4C3T KYVVNNGHTIKVVMGG-RGYVVVDG----KRFYLKQFHFHA------PSEHTVNGKHYPF 104

1KOP VDVENNGHTIQVNYPEGGNTLTVNG----RTYTLKQFHFHV------PSENQIKGRTFPM 105

4G7A SEVVNNGHTIQINIKE-DNTLNYLG----EKYQLKQFHFHT------PSEHTIEKKSYPL 104

4XFW KAVFFTHHTLKASFEP-TNHINYRG----HDYVLDNVHFHA------PMEFLINNKTRPL 105

1CA2 LRILNNGHAFNVEFDDSQDKAVLKGGPLDGTYRLIQFHFHWGSLDGQGSEHTVDKKKYAA 115

: . *::: * : * :.*** * :. :

Ppr_alpha EAHFVHATDK-----------GELAVVAVMFDFGPRSNNELTTLLASIPS-KG-QTVELK 146

4C3T EAHFVHLDKN-----------GNITVLGVFFKVGKE-NPELEKVWRVMPEEPG-QKRHLT 151

1KOP EAHFVHLDEN-----------KQPLVLAVLYEAGKT-NGRLSSIWNVMPMTAG-KV-KLN 151

4G7A EIHFVHKTED-----------GKILVVGVMAKLGKT-NKELDKILNVAPAEEG-EK-ILD 150

4XFW SAHFVHKDAK-----------GRLLVLAIGFEEGKE-NPNLDPILEGIQKKQ------NF 147

1CA2 ELHLVHWNTKYGDFGKAVQQPDGLAVLGIFLKVGSA-KPGLQKVVDVLDSIKTKGKSADF 174

. *:** . *:.: . * : * :

Ppr_alpha EALNPADLLPRDREYYRFNGSLTTPPCSEGVRWFVMQEPQTSSKAQTEKLQAVM------ 200

4C3T ARIDPEKLLPENRDYYRYSGSLTTPPCSEGVRWIVFKEPVEMSREQLEKFRKVM------ 205

1KOP QPFDASTLLPKRLKYYRFAGSLTTPPCTEGVSWLVLKTYDHIDQAQAEKFTRAV------ 205

4G7A KNLNLNNLIPKDKRYMTYSGSLTTPPCTEGVRWIVLKKPISISKQQLEKLKSVM------ 204

4XFW KEVALDAFLPKSINYYHFNGSLTAPPCTEGVAWFVVEEPLEVSAKQLAEIKKRMK----- 202

1CA2 TNFDPRGLLPESLDYWTYPGSLTTPPLLECVTWIVLKEPISVSSEQVLKFRKLNFNGEGE 234

. ::*. * : ****:** * * *:*.: . * ::

Ppr_alpha ----GN-NARPLQPLNARLILE------ 217

4C3T ----GFDNNRPVQPLNARKVMK------ 223

1KOP ----GSENNRPVQPLNARVVIE------ 223

4G7A ----VNPNNRPVQEINSRWIIEGF---- 224

4XFW ----NSPNQRPVQPDYNTVIIKRSAETR 226

1CA2 PEELMVDNWRPAQPLKNRQIKASFK--- 259

* ** *
